# Supplementary material for: Screening the Key Region of Sunlight Regulating the Flavonoid Profiles of Young Shoots in Tea Plants (Camellia sinensis L.) Based on a Field Experiment
Source: Molecules. 2021 Nov 26;26(23):7158. doi: 10.3390/molecules26237158 (PMC8659094; doi:10.3390/molecules26237158)
Supplement: Supplementary file 1 [file molecules-26-07158-s001.zip › molecules-1450926-supplementary.pdf]

Table S1. The contents of catechins in different tea cultivars under shade treatments (mg/g dry weight) [a]

Shade duration	Treatment	GC [b]	EGC [b]	C [b]	EC [b]	EGCG [b]	GCG [b]	ECG [b]	CG [b]	TC
Longjing 43										
No.1	CK	3.26±0.10a	29.51±0.67a	6.82±0.14a	17.15±0.53a	78.37±1.04b	1.06±0.03d	28.64±0.43a	0.50±0.01a	165.31±2.45b
	BN70%	2.02±0.02c	25.38±0.80bc	4.99±0.49b	14.66±0.16b	93.93±0.53a	1.40±0.01b	29.52±0.40a	0.43±0.02b	172.32±0.54a
	BN95%	2.52±0.03b	20.23±1.03d	5.19±0.25b	11.58±0.19d	81.03±1.93b	1.17±0.04c	26.10±1.08b	0.31±0.01c	148.12±4.02c
	BN	2.52±0.09b	26.61±0.06b	5.15±0.32b	13.74±0.20c	94.69±0.56a	1.68±0.04a	24.97±0.20b	0.31±0.02c	169.66±0.56ab
	YN	1.85±0.09c	24.20±1.31c	5.02±0.53b	14.02±0.13bc	92.89±0.64a	1.37±0.02b	29.62±0.32a	0.29±0.02c	169.27±1.34ab
	RN	1.98±0.01c	24.42±0.13bc	5.43±0.05b	14.42±0.17bc	95.01±0.52a	1.35±0.01b	29.55±0.37a	0.29±0.01c	172.45±0.80a
No.2	CK	3.47±0.01a	30.46±1.67a	6.55±0.33a	17.52±0.44a	78.03±0.11c	1.11±0.02d	28.94±0.16ab	0.49±0.00a	166.57±2.04c
	BN70%	2.22±0.13c	27.90±0.03a	5.58±0.13b	15.41±0.03b	95.27±0.42a	1.61±0.02a	28.61±0.13abc	0.51±0.01a	177.10±0.60ab
	BN95%	1.93±0.01d	24.72±0.21b	5.40±0.25ab	12.80±0.49c	90.49±0.45b	1.46±0.03b	27.42±0.15d	0.32±0.00c	164.54±1.16c
	BN	2.18±0.04c	28.77±0.82a	5.88±0.39b	15.31±0.15b	94.81±0.85a	1.39±0.02c	28.16±0.34c	0.33±0.01c	176.84±0.83ab
	YN	2.14±0.08c	29.38±0.24a	5.86±0.09b	15.71±0.08b	91.56±1.06b	1.44±0.02bc	28.39±0.28bc	0.33±0.00c	174.81±1.76b
	RN	2.47±0.04b	29.37±1.79a	5.80±0.15b	16.00±0.31b	95.10±0.56a	1.43±0.02bc	29.03±0.23a	0.36±0.01b	179.57±2.08a
No.3	CK	2.29±0.06a	24.77±0.79a	3.85±0.23ab	10.40±0.09a	89.67±1.38bc	0.97±0.05a	27.06±0.57a	0.50±0.03a	159.52±2.57b
	BN70%	1.45±0.09bc	20.00±0.29c	2.87±0.15c	7.72±0.02c	89.75±1.50bc	0.75±0.01d	23.21±0.24b	0.43±0.01ab	146.17±2.06c
	BN95%	1.27±0.03cd	18.23±0.91d	2.94±0.19c	6.97±0.06e	92.29±0.64b	0.88±0.02b	23.11±0.13b	0.42±0.00bc	146.11±0.12c
	BN	1.13±0.04d	16.92±0.17d	3.43±0.03b	6.40±0.08f	88.52±0.30c	0.89±0.01b	21.88±0.18c	0.35±0.03c	139.52±0.65d
	YN	1.35±0.08bc	22.62±0.47b	3.61±0.21b	7.36±0.05d	91.11±1.71bc	0.78±0.03cd	22.48±0.45bc	0.38±0.04bc	149.68±2.29c
	RN	1.50±0.10b	24.49±0.06a	4.08±0.10a	8.54±0.02b	98.39±0.54a	0.84±0.01bc	26.33±0.28a	0.37±0.03bc	164.53±0.70a
No.4	CK	1.80±0.04a	21.71±0.16c	3.41±0.25c	2.23±0.05d	80.76±0.06d	0.84±0.01d	23.40±0.05a	0.51±0.01b	134.65±0.12c
	BN70%	1.33±0.03cd	21.09±1.65c	3.66±0.27bc	8.25±0.55b	91.61±0.97b	0.85±0.03d	20.78±0.13c	0.40±0.02c	147.97±3.16b
	BN95%	1.22±0.04d	14.62±0.11d	3.49±0.19c	6.11±0.48c	85.87±0.56c	1.00±0.02c	19.60±0.15d	0.36±0.03c	132.26±0.48c
	BN	1.56±0.06b	25.71±1.94b	4.86±0.36a	10.02±0.11a	99.33±3.79a	3.01±0.06a	20.31±0.22c	0.76±0.01a	163.99±1.76a
	YN	1.29±0.06d	28.87±0.21a	3.54±0.26bc	10.04±0.21a	99.47±0.92a	1.16±0.03b	22.73±0.37b	0.39±0.02c	167.50±1.04a
	RN	1.45±0.07bc	27.28±0.32ab	4.18±0.08ab	9.97±0.10a	99.76±0.85a	1.05±0.01c	22.58±0.27b	0.37±0.01c	166.64±1.10a

No.5	CK	1.95±0.04b	23.71±0.35e	4.38±0.19a	11.26±0.04a	72.09±0.34d	0.96±0.02d	23.72±0.18a	0.46±0.02a	138.53±0.96e
	BN70%	2.06±0.02b	31.79±0.27b	3.53±0.39b	10.32±0.11b	90.81±0.63b	0.97±0.03cd	21.17±0.13c	0.26±0.01b	160.91±1.22c
	BN95%	1.59±0.04d	17.50±0.08f	2.32±0.15c	6.14±0.08d	78.39±0.50c	1.04±0.04c	16.92±0.14d	0.20±0.00c	124.10±0.79f
	BN	2.27±0.05a	25.07±0.47d	3.90±0.30ab	9.31±0.13c	89.27±1.30b	1.31±0.01a	21.13±0.34c	0.21±0.00c	152.48±2.29d
	YN	1.82±0.02c	29.96±0.05c	3.55±0.20b	10.54±0.08b	95.19±0.27a	1.29±0.01a	24.03±0.07a	0.21±0.00c	166.59±0.52b
	RN	2.21±0.09a	33.55±0.12a	3.73±0.05ab	11.07±0.02a	96.08±0.36a	1.17±0.02b	22.62±0.20b	0.26±0.01b	170.68±0.61a
Zhongming 192										
No.1	CK	6.07±0.35a	59.74±0.65bc	2.81±0.82a	18.83±0.16ab	94.32±1.06c	1.24±0.06a	19.53±0.33a	0.54±0.03a	203.08±1.77b
	BN70%	5.83±0.29a	62.14±1.73ab	2.81±0.33a	19.32±0.43a	96.35±2.47bc	1.27±0.04a	19.62±0.48a	0.49±0.01bc	207.82±5.18ab
	BN95%	5.70±0.23a	56.45±0.74d	2.38±0.24a	16.98±0.48d	92.96±0.69c	1.22±0.05a	18.30±0.16b	0.49±0.01bc	194.47±1.98c
	BN	5.74±0.23a	57.96±0.87cd	2.88±0.33a	17.87±0.34c	98.49±1.36ab	1.37±0.04a	19.39±0.25a	0.47±0.01c	204.18±3.29ab
	YN	5.30±0.20a	61.63±0.48ab	2.31±0.13a	18.45±0.20abc	101.48±1.07a	1.21±0.06a	19.62±0.24a	0.48±0.02bc	210.48±1.66ab
	RN	5.36±0.36a	63.07±1.00a	2.55±0.09a	18.11±0.14bc	101.76±0.90a	1.26±0.10a	18.85±0.16ab	0.52±0.01ab	211.46±1.80a
No.2	CK	4.93±0.37a	51.01±1.22cd	3.49±0.26a	18.42±0.33a	110.99±2.62bc	1.59±0.05b	22.54±0.64b	0.55±0.02b	213.52±4.38a
	BN70%	5.10±0.19a	56.01±1.10a	2.47±0.12b	16.32±0.43cd	98.69±1.24d	1.26±0.08d	18.99±0.28cd	0.42±0.02e	199.26±2.61b
	BN95%	5.08±0.49a	55.27±0.70ab	2.32±0.23b	15.70±0.19d	101.22±1.44d	1.28±0.03cd	18.20±0.28d	0.44±0.01de	199.52±3.10b
	BN	4.53±0.30ab	56.58±0.81a	2.50±0.50b	16.34±0.23cd	107.07±0.50c	1.36±0.05cd	19.71±0.12c	0.47±0.01cd	208.57±1.29ab
	YN	4.88±0.12a	49.67±1.73d	2.73±0.10ab	17.55±0.59ab	113.62±3.21ab	1.80±0.06a	24.13±0.74a	0.68±0.03a	215.07±6.47a
	RN	3.90±0.19b	52.84±0.91bc	2.50±0.38b	17.00±0.29bc	117.41±1.49a	1.42±0.03c	21.85±0.30b	0.51±0.01bc	217.43±3.01a
No.3	CK	4.03±0.18a	48.74±0.55a	2.96±1.04a	19.53±0.18a	126.71±0.72c	1.59±0.02b	24.08±0.47ab	0.43±0.02c	228.07±2.47ab
	BN70%	2.92±0.17bc	40.54±0.40d	2.68±0.27a	16.50±0.18cd	132.66±1.11b	1.73±0.02a	24.22±0.06ab	0.63±0.01b	221.87±1.30bc
	BN95%	2.57±0.07d	39.84±0.93d	2.50±0.24a	15.53±0.17e	131.93±3.27b	1.61±0.05b	23.17±0.59b	0.62±0.03b	217.76±5.24c
	BN	2.82±0.10cd	43.38±0.34c	2.46±0.15a	16.30±0.05d	138.66±2.12a	1.55±0.02bc	24.56±0.34a	0.69±0.01a	230.44±2.93a
	YN	3.26±0.10b	46.45±0.21b	2.68±0.19a	18.40±0.13b	136.67±0.71ab	1.57±0.02b	24.59±0.14a	0.61±0.01b	234.23±0.86a
	RN	2.88±0.08cd	47.30±1.16ab	2.22±0.35a	17.00±0.34c	138.19±1.63a	1.49±0.03c	23.59±0.63ab	0.69±0.00a	233.37±3.72a
No.4	CK	3.87±0.13a	55.58±3.88a	2.36±0.11a	17.14±0.29a	115.49±1.89d	1.43±0.03b	20.82±0.35c	0.63±0.02b	217.31±6.05b
	BN70%	2.14±0.04c	36.57±0.64c	2.04±0.12ab	10.54±0.28c	125.08±2.22c	4.88±0.11a	21.12±0.47bc	0.69±0.02a	202.67±2.83c
	BN95%	3.04±0.06b	46.48±0.28b	1.79±0.10bc	14.14±0.44b	126.89±0.84c	1.47±0.00b	19.80±0.10d	0.56±0.01c	214.17±1.67b

	BN	3.62±0.16a	55.04±1.09a	1.45±0.03c	16.78±0.53a	129.39±2.17bc	1.48±0.07b	21.14±0.34bc	0.57±0.01c	229.47±4.10a
	YN	3.90±0.16a	57.33±0.39a	1.56±0.24c	17.21±0.22a	134.54±1.41a	1.35±0.02b	22.20±0.27a	0.57±0.00c	238.66±2.14a
	RN	3.59±0.20a	55.98±0.53a	1.59±0.08c	17.30±0.58a	132.00±1.59ab	1.39±0.07b	21.84±0.27ab	0.56±0.01c	234.24±2.73a
No.5	CK	4.90±0.05a	56.76±1.76ab	1.70±0.10a	16.84±0.14a	99.05±2.26d	1.25±0.03a	19.30±0.54b	0.55±0.04a	200.36±4.60cd
	BN70%	3.99±0.13bc	58.22±0.28ab	1.38±0.12ab	14.66±0.06bc	111.67±0.49b	1.13±0.06a	19.71±0.24b	0.47±0.02b	211.21±0.53ab
	BN95%	3.20±0.19d	52.78±0.71c	0.91±0.18c	13.12±0.48d	105.26±0.52c	1.14±0.02a	17.30±0.40c	0.49±0.03ab	194.20±2.05d
	BN	3.79±0.11c	55.81±0.67b	1.34±0.27ab	14.90±0.09b	117.00±1.23a	1.27±0.02a	20.61±0.23a	0.49±0.02ab	215.20±2.44a
	YN	3.99±0.26bc	58.49±0.30a	1.06±0.09bc	14.13±0.15c	107.57±0.85c	1.15±0.05a	17.40±0.11c	0.38±0.02c	204.18±1.26bc
	RN	4.37±0.15b	58.88±0.68a	0.83±0.08c	15.37±0.38b	106.63±2.24c	1.27±0.10a	15.73±0.14d	0.36±0.01c	203.44±2.71c
Wanghai 1										
No.1	CK	3.64±0.13a	38.91±0.98b	3.76±0.42a	18.96±0.33b	95.69±3.51ab	1.55±0.02a	30.79±1.14a	0.78±0.19a	194.07±5.19a
	BN70%	3.67±0.23a	45.28±2.04a	3.39±0.22abc	21.43±1.39a	95.01±3.24ab	1.23±0.05b	25.80±0.71c	0.52±0.01b	196.33±7.19a
	BN95%	2.61±0.06c	32.87±1.02c	3.16±0.06abc	15.15±0.18c	84.43±4.84b	1.05±0.03d	23.88±0.40d	0.46±0.01b	163.62±3.97b
	BN	3.04±0.25bc	40.92±0.20b	3.03±0.20bc	19.33±0.35b	95.46±0.80ab	1.12±0.03cd	25.65±0.49c	0.51±0.01b	189.07±1.05a
	YN	3.11±0.10b	42.01±0.48b	2.74±0.23c	21.06±0.10a	98.23±0.49a	1.15±0.00bc	27.61±0.16b	0.45±0.02b	196.36±1.12a
	RN	3.31±0.12ab	40.47±1.16b	3.43±0.19ab	19.87±0.36ab	94.96±7.47ab	1.23±0.02b	27.10±0.26bc	0.42±0.04b	190.78±7.10a
No.2	CK	3.49±0.11a	34.58±0.56c	3.84±0.02ab	19.01±0.76b	91.91±1.40cd	1.53±0.02a	29.16±0.43b	0.68±0.02a	184.19±3.24d
	BN70%	3.60±0.05a	41.32±0.69b	4.27±0.29a	21.61±0.09a	99.49±1.64b	1.39±0.02b	28.38±0.49b	0.55±0.01bc	200.61±2.50bc
	BN95%	2.65±0.04d	32.58±0.71c	3.20±0.30b	15.58±0.44c	88.40±1.66d	0.98±0.01d	25.49±0.64c	0.52±0.01c	169.39±3.67e
	BN	3.21±0.04b	43.66±0.88a	3.52±0.09b	21.64±0.73a	106.80±2.20a	1.38±0.02b	31.70±0.65a	0.54±0.02bc	212.45±4.31a
	YN	2.97±0.10c	39.79±0.99b	2.92±0.21c	19.31±0.83b	97.14±4.03bc	1.19±0.05c	27.46±0.98b	0.57±0.02b	191.36±6.75cd
	RN	2.97±0.08c	39.04±1.05b	3.30±0.45b	20.44±0.76ab	108.97±0.46a	1.33±0.05b	32.42±0.64a	0.55±0.01bc	209.03±2.81ab
No.3	CK	2.33±0.09a	35.67±0.61b	2.36±0.06ab	17.20±0.26e	79.24±1.32d	1.19±0.06d	22.05±0.28c	0.72±0.03a	160.74±2.46c
	BN70%	2.17±0.06a	36.98±0.35b	2.45±0.21a	19.19±0.30c	86.54±1.27b	1.26±0.01cd	23.03±0.36b	0.63±0.02b	172.26±2.43b
	BN95%	1.75±0.15b	30.71±0.47c	2.04±0.08b	14.74±0.32f	82.31±1.46cd	1.32±0.04bc	20.39±0.35d	0.48±0.01d	153.73±2.75d
	BN	2.11±0.11a	35.95±0.46b	2.22±0.03ab	18.08±0.20d	85.12±1.05bc	1.31±0.04bc	21.98±0.25c	0.60±0.02b	167.37±2.04b
	YN	2.13±0.03a	40.73±0.70a	2.29±0.20ab	20.88±0.15a	94.47±1.34a	1.41±0.02ab	25.42±0.36a	0.51±0.01cd	187.85±2.55a
	RN	2.29±0.10a	40.63±0.38a	2.16±0.03ab	19.98±0.17b	95.28±0.30a	1.46±0.06a	23.80±0.07b	0.53±0.01c	186.13±0.58a

No.4	CK	2.88±0.03a	41.97±4.86bc	2.66±0.05a	18.55±0.15a	80.58±0.53a	1.19±0.03a	23.01±0.15a	0.63±0.01a	171.47±5.37a
	BN70%	1.93±0.05b	47.82±1.31a	1.96±0.14b	16.31±0.64c	72.63±2.59b	1.04±0.02a	17.68±0.68b	0.60±0.02a	159.96±5.14bc
	BN95%	1.42±0.07c	32.70±0.32d	1.34±0.06c	10.11±0.15d	68.06±0.82c	1.16±0.02a	15.83±0.18c	0.45±0.01c	131.07±1.43d
	BN	1.74±0.17b	36.74±0.84cd	1.87±0.11b	15.33±0.35c	76.34±1.86ab	1.02±0.22a	18.25±0.51b	0.51±0.02b	151.78±1.10c
	YN	1.76±0.05b	42.61±0.10ab	1.89±0.16b	17.55±0.36b	79.22±2.84a	1.03±0.22a	18.67±0.15b	0.45±0.00c	163.17±3.84ab
	RN	1.81±0.09b	43.47±0.29ab	1.86±0.17b	18.19±0.27ab	79.04±0.43a	1.08±0.06a	18.51±0.12b	0.48±0.00bc	164.43±0.89ab
No.5	CK	3.39±0.08a	42.74±2.54b	2.13±0.09a	19.98±1.00a	72.48±4.52ab	0.93±0.02a	22.56±1.23a	0.71±0.10a	164.92±9.41a
	BN70%	2.45±0.21b	43.94±0.99c	1.26±0.14bc	15.15±0.07b	64.83±0.75cd	0.81±0.03a	14.66±0.29c	0.44±0.04b	143.54±1.55b
	BN95%	1.18±0.17c	23.65±0.64b	0.73±0.09d	7.60±0.20c	59.53±1.64d	0.70±0.02ba	12.39±0.37d	0.35±0.05b	106.13±2.96c
	BN	2.43±0.22b	44.34±0.44b	1.48±0.05b	15.82±0.21b	70.19±0.51bc	0.84±0.00a	17.20±0.24b	0.46±0.01b	152.76±1.15ab
	YN	2.18±0.06b	48.73±0.15a	1.08±0.08cd	15.61±0.09b	62.46±4.18d	0.54±0.24b	15.01±0.10c	0.33±0.02b	145.92±4.17b
	RN	2.48±0.16b	47.72±0.49a	1.36±0.28bc	16.35±0.26b	77.62±0.81a	0.95±0.02a	16.67±0.24b	0.44±0.01b	163.59±1.47a
Jingning 1										
No.1	CK	3.28±0.68a	33.17±1.03ab	3.45±0.03a	13.86±0.10a	137.80±0.97a	1.47±0.05a	36.80±0.46a	1.26±0.05a	231.10±2.05a
	BN70%	2.95±0.08a	31.73±0.39bc	2.48±0.25b	13.03±0.12b	136.21±0.92a	1.43±0.01ab	33.32±0.22b	1.01±0.01b	222.16±1.58ab
	BN95%	3.15±0.11a	33.80±0.84a	2.69±0.03b	12.31±0.38c	134.00±3.59a	1.26±0.02cd	31.49±0.89c	0.96±0.02bc	219.67±5.85bc
	BN	3.07±0.09a	25.27±0.24d	2.59±0.28b	11.49±0.29d	133.58±2.18a	1.32±0.03bcd	32.90±0.60bc	0.89±0.04c	211.12±2.67c
	YN	3.21±0.28a	30.46±0.30c	2.41±0.27b	12.18±0.22c	138.76±3.21a	1.38±0.05abc	33.41±0.82b	0.94±0.04bc	222.76±3.85ab
	RN	2.95±0.31a	26.02±0.18d	2.17±0.13b	10.85±0.13e	138.39±1.22a	1.24±0.09d	34.41±0.43b	1.01±0.02b	217.03±1.40bc
No.2	CK	3.35±0.16ab	26.08±0.31ab	2.20±0.22ab	13.02±0.19a	138.13±2.28d	1.35±0.03a	37.95±0.62a	1.37±0.05b	223.47±3.18bc
	BN70%	3.13±0.07ab	27.05±0.25a	2.32±0.05a	12.78±0.07a	144.60±1.27b	0.48±0.05bc	36.52±0.28ab	1.25±0.03b	228.12±1.81ab
	BN95%	3.69±0.86a	22.60±0.25c	2.17±0.02ab	10.62±0.07c	139.71±0.69cd	1.13±0.60ab	33.32±0.15d	1.95±0.03a	215.19±0.40d
	BN	2.63±0.25b	25.54±0.41b	2.15±0.23ab	11.59±0.19b	143.37±1.38bc	0.40±0.03c	33.67±0.42cd	1.26±0.01b	220.60±2.42cd
	YN	2.98±0.07ab	26.98±0.63a	1.77±0.19b	12.50±0.41a	149.90±2.12a	0.42±0.01c	35.82±0.33abc	1.33±0.04b	231.69±3.28a
	RN	2.66±0.01b	26.23±0.36ab	2.04±0.24ab	11.44±0.53bc	148.11±2.09ab	0.43±0.01c	34.39±1.86bcd	1.22±0.23b	226.51±0.44abc
No.3	CK	2.53±0.09a	25.01±0.16c	2.39±0.07ab	12.03±0.03a	138.98±1.14a	1.13±0.00a	30.59±0.17a	1.03±0.01a	213.69±1.45a
	BN70%	2.32±0.16ab	23.88±0.15d	2.37±0.21ab	11.03±0.09c	134.50±1.73bc	1.00±0.01b	27.02±0.29b	0.98±0.02b	203.09±2.18c
	BN95%	2.03±0.31b	20.96±0.11d	2.13±0.12b	9.42±0.07e	132.88±1.51c	0.86±0.00d	25.31±0.21d	0.84±0.01d	194.42±1.49d

	BN	2.16±0.19ab	24.72±0.08c	2.59±0.11ab	10.44±0.09d	126.98±0.86d	0.96±0.00c	23.90±0.25e	0.90±0.01c	192.63±1.33d
	YN	2.30±0.12ab	26.37±0.03b	2.64±0.23ab	11.63±0.08b	136.91±0.93ab	1.00±0.03b	26.02±0.31c	0.88±0.01cd	207.75±1.55bc
	RN	2.40±0.05ab	27.96±0.43a	2.69±0.31a	11.64±0.19b	136.49±1.75abc	1.02±0.02b	25.74±0.27cd	1.01±0.02ab	208.96±2.67ab
No.4	CK	2.34±0.02bc	32.55±0.18a	3.07±0.13a	12.08±0.21b	128.82±1.00bc	0.91±0.02e	25.73±0.14b	0.91±0.02a	206.41±0.99a
	BN70%	2.65±0.21ab	33.40±0.30a	2.52±0.23b	11.08±0.13c	136.53±0.39ab	0.96±0.01d	24.51±0.17c	0.84±0.01b	212.49±0.86a
	BN95%	1.95±0.49cd	26.00±0.08b	2.08±0.14bc	9.00±0.31d	130.82±0.45bc	1.01±0.01c	22.80±0.08d	0.63±0.00d	194.30±0.49b
	BN	3.23±0.00a	32.75±3.52a	1.94±0.03c	16.29±0.09a	133.37±0.97ab	1.12±0.00a	22.95±0.20d	0.59±0.01e	212.24±2.79a
	YN	2.23±0.08bcd	32.61±0.16a	2.26±0.29bc	11.08±0.03c	140.81±1.54a	1.07±0.01b	24.51±0.35c	0.68±0.01c	215.24±2.35a
	RN	1.70±0.01d	23.21±0.12b	2.14±0.04bc	11.30±0.11c	123.15±7.57c	0.57±0.01f	27.32±0.20a	0.35±0.01f	189.73±7.63b
No.5	CK	2.58±0.11bc	33.63±1.44a	3.22±0.14a	13.33±0.01a	123.67±1.64d	0.83±0.01d	28.23±0.47b	0.80±0.01ab	206.30±3.59bc
	BN70%	3.15±0.19a	29.95±0.28b	2.72±0.12b	11.79±0.15b	133.39±1.01b	1.21±0.02b	27.33±0.19c	0.68±0.00bc	210.22±1.65b
	BN95%	2.34±0.24c	29.67±0.23b	1.98±0.20cd	9.90±0.12d	129.56±0.83c	1.13±0.01c	27.01±0.26c	0.94±0.19a	202.53±1.56c
	BN	1.70±0.18d	30.11±0.29b	1.60±0.08d	8.02±0.18f	120.29±1.52d	1.23±0.03b	22.26±0.33d	0.56±0.02c	185.76±2.43e
	YN	1.90±0.11d	34.63±0.26a	1.81±0.21d	8.84±0.05e	122.07±0.53d	1.28±0.01a	22.14±0.10d	0.56±0.03c	193.23±0.96d
	RN	2.89±0.06ab	30.43±0.28b	2.38±0.26bc	10.93±0.28c	138.63±1.48a	1.23±0.01b	30.29±0.33a	0.91±0.02a	217.68±1.81a
Zhonghuang 2										
No.1	CK	2.45±0.03a	5.51±0.05cd	2.89±0.04a	15.82±0.27a	104.56±3.09b	1.08±0.02ab	23.88±0.64a	0.49±0.06a	156.67±3.45b
	BN70%	2.48±0.39a	6.05±0.17b	2.49±0.13b	13.91±0.74b	114.54±0.86a	1.03±0.07bc	23.24±1.80ab	0.45±0.03ab	164.19±3.40ab
	BN95%	2.09±0.29a	5.34±0.09d	2.34±0.17b	12.72±0.13d	102.56±0.34b	1.00±0.02bc	21.08±0.08b	0.42±0.01ab	147.55±0.48c
	BN	2.58±0.50a	5.69±0.06c	2.65±0.15ab	13.75±0.16bc	107.24±3.54b	0.99±0.01c	23.05±0.67ab	0.37±0.02b	156.31±5.02b
	YN	2.70±0.15a	6.23±0.13b	2.60±0.03ab	14.37±0.14b	114.70±0.89a	1.01±0.02bc	23.94±0.31a	0.41±0.01b	165.97±1.28a
	RN	2.39±0.29a	6.87±0.05a	1.64±0.10c	12.95±0.13cd	114.36±1.70a	1.12±0.02a	22.91±0.29ab	0.50±0.02a	162.74±2.10ab
No.2	CK	2.99±0.13a	33.15±0.21ab	2.06±0.13a	13.77±0.17a	105.82±0.51c	1.08±0.00b	24.59±0.10b	0.50±0.01ab	183.97±0.83b
	BN70%	2.39±0.29a	33.76±0.31a	2.21±0.18a	14.17±0.44a	121.08±2.09a	1.15±0.00a	26.04±0.60a	0.54±0.01a	201.35±2.16a
	BN95%	2.63±0.25a	25.52±0.16e	2.01±0.21a	12.24±0.21b	115.56±0.79ab	1.05±0.01b	23.74±0.18bc	0.46±0.01c	183.21±1.13b
	BN	2.55±0.22a	28.23±0.52d	2.32±0.09a	13.61±0.13a	112.52±1.57b	1.08±0.02b	23.05±0.35c	0.46±0.01bc	183.83±2.53b
	YN	2.54±0.52a	31.20±1.30c	2.29±0.29a	14.44±0.54a	116.66±4.48ab	0.50±0.02d	24.06±0.94bc	0.49±0.03bc	192.18±8.06ab
	RN	2.52±0.35a	31.90±0.37bc	1.99±0.13a	14.39±0.21a	117.33±0.92ab	0.54±0.02c	23.75±0.08bc	0.48±0.00bc	192.91±1.45ab

No.3	CK	1.34±0.10a	26.04±0.25a	1.89±0.20a	11.03±0.41d	94.67±0.62c	1.72±0.02c	18.32±0.14d	0.48±0.01b	155.49±0.93e
	BN70%	1.23±0.05a	24.21±0.40b	1.68±0.17ab	12.80±0.31ab	115.12±1.60a	2.33±0.05a	22.11±0.31a	0.51±0.01b	179.99±2.74d
	BN95%	1.23±0.05a	21.22±0.19b	1.87±0.01a	12.11±0.11c	107.05±1.09b	2.00±0.04b	18.99±0.19c	0.48±0.01a	164.96±1.22a
	BN	1.27±0.02a	24.15±0.33c	1.45±0.16b	12.56±0.14bc	113.20±1.29a	1.97±0.03b	20.00±0.19b	0.49±0.01b	175.09±2.07ab
	YN	1.23±0.06a	26.79±0.44a	1.49±0.05b	13.22±0.26ab	105.19±1.60b	1.47±0.04d	18.34±0.31d	0.46±0.01c	168.20±2.48cd
	RN	1.18±0.05a	26.21±0.23a	1.57±0.06ab	13.37±0.12a	108.35±0.86b	1.80±0.04c	18.55±0.10cd	0.50±0.01ab	171.54±1.35bc
No.4	CK	1.64±0.02a	31.91±0.17c	1.60±0.09a	11.06±0.13ab	101.18±1.12d	2.34±0.03a	19.45±0.23c	0.27±0.01a	169.46±1.63c
	BN70%	1.54±0.13ab	33.73±0.89b	1.19±0.02b	11.48±1.15a	108.44±2.05bc	2.35±0.07a	20.59±0.35b	0.17±0.01b	179.49±3.58ab
	BN95%	1.14±0.06d	28.57±0.86d	1.22±0.06b	9.49±0.50b	105.30±0.90c	1.85±0.02d	19.22±0.16c	0.16±0.01b	166.94±1.84c
	BN	1.32±0.06cd	31.91±0.59c	1.34±0.09b	11.55±0.43a	109.71±1.00b	2.04±0.02c	20.24±0.23b	0.18±0.03b	178.28±1.64b
	YN	1.30±0.04cd	32.25±0.46bc	1.29±0.09b	11.12±0.24a	115.88±2.04a	2.17±0.05b	22.03±0.42a	0.17±0.02b	186.21±3.12a
	RN	1.40±0.10bc	41.28±0.52a	1.17±0.02b	11.93±0.43a	105.95±1.32bc	1.65±0.02e	17.16±0.23d	0.18±0.01b	180.72±2.29ab
No.5	CK	3.09±0.05a	32.88±0.92cd	1.64±0.22a	11.85±0.05c	107.48±1.29a	2.04±0.02a	24.91±0.31a	0.43±0.00a	184.32±0.51a
	BN70%	2.09±0.18b	39.72±0.51a	1.31±0.19a	12.97±0.20a	89.19±1.92c	1.30±0.05d	21.98±0.46b	0.36±0.03ab	168.92±2.92b
	BN95%	2.33±0.13b	32.40±2.08d	1.27±0.14a	11.02±0.05d	79.98±0.52d	1.03±0.03e	18.35±0.22d	0.32±0.05ab	146.70±1.90d
	BN	2.61±0.19ab	35.55±1.45bcd	1.39±0.10a	12.24±0.34bc	108.09±3.56a	1.88±0.08b	25.88±0.80a	0.36±0.01ab	188.01±4.15a
	YN	2.49±0.30b	36.62±2.14abc	1.40±0.02a	12.14±0.07bc	82.93±1.00d	1.03±0.01e	18.70±0.27d	0.25±0.02b	155.55±3.17c
	RN	2.27±0.25b	38.77±1.00ab	1.45±0.05a	12.47±0.04b	96.22±0.89b	1.52±0.03c	20.69±0.25c	0.36±0.12ab	173.75±0.87b

[a] Abbreviation: EC: (−)-epicatechin; EGC: (−)-epigallocatechin; ECG: (−)-epicatechin gallate; EGCG: (−)-epigallocatechin gallate; GC: (+)-gallocatechin; C: (+)-catechin; TC: Total catechins; BN70%: samples under black net 70% shade treatment; BN95%: samples under black net 95% shade treatment; BN: samples under blue net shade treatment; YN: samples under yellow net shade treatment; RN: samples under red net shade treatment. For the same tea cultivars with the same serial number, data with different alphabetic letters (a, b, c, d, e, f) in the same column were significantly different at $P<0.05$. Significant difference analysis was carried out by the SAS System for Windows version 8.1 (SAS Institute Inc., Cary, NC, USA) using Tukey test. Data expressed as mean±SD represent n = 3 replicates.

[b] Quantified by the corresponding authentic standards.

Table S2 The contents of flavonol glycosides in different tea cultivars under shade treatments (μg/g dry weight) [a]

Time	Treatment	M-gal-rha-glu [b]	M-gal [b]	M-glu [b]	Q-gal-rha-glu [b]	Q-glu-rha-glu [b]	Q-glu-rha-rha [b]	Q-glu-rha [b]	Q-gal [b]	Q-glu [b]	K-glu-rha-glu [b]	K-gal [b]	K-glu-rha [b]	K-glu [b]	TFG
Longjing 43															
No.1	CK	69±2a	953±33a	479±9a	470±13a	358±20a	746±9a	193±19a	385±5a	100±5a	302±8a	324±22a	75±10a	36±3a	4490±121a
	BN70%	54±4b	591±18b	214±7b	210±1b	115±5b	784±37a	84±8b	166±5b	35±3b	237±9b	330±9a	48±2b	34±1a	2902±50b
	BN95%	34±1d	413±7e	137±5c	152±5c	84±3c	704±33a	68±5bc	116±3c	14±1c	152±9c	269±12ab	42±7b	20±1c	2205±8c
	BN	35±1d	423±3de	137±2c	161±3c	73±2c	773±97a	55±0c	95±1d	7±1d	151±7c	215±26b	39±2b	20±1bc	2186±80c
	YN	38±1d	474±1c	136±3c	129±4d	69±1c	668±56a	64±10bc	120±3c	10±1cd	142±9c	312±48a	53±5b	24±1b	2240±105c
	RN	46±2c	467±12cd	139±9c	132±1d	61±1c	680±43a	61±7bc	119±4c	12±1cd	157±6c	269±1ab	37±8b	22±1bc	2201±49c
No.2	CK	68±1a	1105±29a	551±6a	549±4a	487±9a	717±43b	195±33a	490±8a	144±6a	314±4a	296±40a	72±16a	42±4a	5029±101a
	BN70%	59±2b	726±47b	260±4b	248±1b	121±4b	936±54a	71±5b	194±3b	46±5b	293±11a	343±17a	66±7a	36±3a	3398±127b
	BN95%	40±2d	453±5d	147±3d	168±8c	63±1d	886±41a	53±14b	127±7d	18±0cd	184±1c	291±17a	56±9a	26±0b	2513±69cd
	BN	44±3d	486±11d	153±3d	145±6d	75±3c	815±65ab	45±10b	113±1e	16±0d	168±9c	271±19a	48±8a	24±2b	2403±42d
	YN	34±0e	452±5d	127±4e	139±3d	74±2cd	822±60ab	42±1b	111±3e	15±1d	172±2c	300±28a	61±2a	26±2b	2375±81d
	RN	51±1c	580±11c	167±7c	166±4c	70±2cd	818±77ab	52±4b	144±2c	28±5c	207±12b	298±29a	56±8a	28±1b	2664±114c
No.3	CK	61±2a	1151±24a	474±10a	476±15a	371±9a	806±32ab	135±6a	394±10a	78±3a	308±13a	294±26a	64±9a	42±3a	4653±89a
	BN70%	53±3b	611±7b	201±8b	231±4b	90±4bc	892±12a	64±5b	145±6b	37±2b	275±12b	254±25ab	32±7b	24±2b	2908±44b
	BN95%	52±6b	481±3d	146±5d	167±2c	90±2bc	799±50ab	41±8cd	105±5d	25±3d	207±9c	186±31c	25±8b	18±1c	2341±78d
	BN	40±1d	412±5e	126±3e	130±3d	77±1d	748±42b	32±3d	81±3e	9±1e	185±2d	147±13c	20±3b	13±1d	2021±34e
	YN	42±2cd	460±6d	133±4de	139±3d	82±1cd	785±61ab	41±5cd	94±3de	29±0cd	218±5cd	202±13bc	20±0b	16±2cd	2263±55d
	RN	49±1bc	556±9c	166±6c	182±3c	99±1b	890±16a	51±5bc	124±2c	33±1bc	254±3b	290±24a	25±5b	24±1b	2743±13c
No.4	CK	76±7b	1217±1a	748±1a	618±3a	613±2a	759±3b	241±16a	528±8a	172±2a	294±6b	250±21a	67±8a	44±1b	5628±51a
	BN70%	57±0bc	582±4b	285±7b	240±2b	161±5c	895±93a	73±12bc	166±6b	55±2c	223±8c	165±12bc	31±1c	20±1c	2954±75b
	BN95%	33±2c	373±6d	149±8e	152±3d	104±0d	717±28b	33±3d	106±5d	34±1d	166±3d	127±17c	20±3d	14±1e	2026±54d
	BN	121±18a	277±1e	227±4c	41±0f	249±3b	258±11c	88±21b	102±6d	97±5b	493±16a	216±46ab	43±3b	68±2a	2278±95d
	YN	48±10c	367±7d	129±9f	114±2e	75±3e	941±28a	36±5d	90±7d	27±2e	162±13d	172±15bc	27±2cd	15±1de	2205±50e
	RN	42±8c	446±8c	167±5d	163±2c	103±3d	946±13a	47±5cd	131±5c	37±2d	174±8d	197±16ab	25±2cd	18±1cd	2495±19c

No.5	CK	41±0a	991±14a	1016±10a	749±6a	1066±5a	606±43ab	464±20a	566±6a	316±5a	268±2a	159±16a	83±8a	44±2a	6370±48a
	BN70%	24±1c	527±8b	331±5b	331±4b	258±7b	549±19ab	87±12bc	175±5b	66±2b	125±2b	78±4cd	20±7bc	15±0b	2586±54b
	BN95%	16±0e	183±2f	138±6c	118±2d	97±4c	500±25b	52±1c	59±1e	15±2d	83±4d	71±17d	15±2c	9±1d	1357±20e
	BN	19±1d	263±9d	139±7c	97±3e	60±1e	649±40a	63±13bc	62±4e	10±1d	83±1d	105±5bc	26±3bc	11±1cd	1587±20d
	YN	34±2b	222±5e	93±3d	117±3d	77±3d	652±65a	71±8bc	78±3d	11±3d	94±1c	117±12b	25±6bc	12±0bcd	1603±68d
	RN	32±0b	300±2c	154±2c	148±0c	101±5c	653±23a	61±3bc	94±4c	32±1c	128±4b	110±6b	32±5b	13±1bc	1858±31c
Zhongming 192															
No.1	CK	50±3a	929±2a	647±9a	726±10a	1225±14a	1102±99b	477±14a	713±12a	242±5a	507±11bc	547±25ab	159±12a	87±3ab	7412±156a
	BN70%	52±3a	804±10b	475±8b	648±19b	984±20b	1054±31b	315±54b	574±10b	170±4b	509±9bc	559±41ab	124±2bc	59±3c	6326±116b
	BN95%	47±4a	691±7c	432±2c	593±2c	959±49b	1138±120ab	317±43b	442±5c	154±4c	523±5ab	525±10b	120±4c	63±2c	6004±201bc
	BN	47±2a	706±9c	412±5d	527±3d	805±10d	1135±81ab	250±11b	446±8c	142±3d	495±5c	496±23b	115±8c	66±6c	5643±61d
	YN	51±4a	706±15c	409±9d	544±7d	877±10c	1095±122b	250±4b	442±4c	143±3d	524±7ab	566±33ab	123±21bc	83±4b	5811±98cd
	RN	54±2a	606±10d	376±3e	539±5d	717±4e	1394±91a	262±42b	434±2c	128±1e	543±11a	677±99a	152±5ab	95±6a	5978±17cd
No.2	CK	53±1a	946±28a	590±7a	694±20a	874±40a	1198±151ab	310±58a	811±29a	227±9a	608±11a	733±20a	189±19a	123±9a	7355±57a
	BN70%	46±3b	734±11b	381±6b	531±13b	661±4b	968±63b	238±17ab	498±9b	118±2b	418±11d	546±68bc	117±15b	64±2c	5321±167b
	BN95%	41±2bc	518±20d	286±6d	395±6d	454±17c	1007±53ab	159±18b	308±14d	101±6c	494±16c	480±19c	89±8b	61±1c	4394±122d
	BN	38±2c	632±41c	355±2c	448±9c	486±8c	1037±87ab	210±44b	370±5c	127±4b	518±1bc	556±49bc	92±15b	63±1c	4933±79c
	YN	45±3b	626±27c	275±12d	360±11e	301±10e	1198±67ab	155±23b	314±10d	88±6c	527±9b	604±78abc	97±2b	82±4b	4673±172cd
	RN	40±2bc	604±45c	281±2d	387±4d	387±8d	1232±65a	198±28b	328±3d	101±2c	530±7b	672±58ab	161±22a	86±5b	5009±172bc
No.3	CK	66±10a	994±6a	568±6a	738±10a	701±11a	1452±94a	202±3a	696±9a	217±5a	771±2a	1011±176a	170±50a	117±9a	7702±328a
	BN70%	48±3b	733±8b	307±2b	477±14b	355±6b	1412±6a	116±6b	380±3b	98±1b	646±20c	833±71ab	145±30a	105±3ab	5653±108b
	BN95%	55±5ab	558±8d	241±10d	341±10c	222±8c	1461±140a	74±26dc	240±7c	67±1cd	607±20cd	780±108ab	134±6a	81±10c	4863±141d
	BN	58±3ab	635±15c	278±6c	356±4c	221±8c	1681±38a	92±8bc	243±4c	77±2c	711±15ab	906±24ab	143±9a	95±3bc	5495±48bc
	YN	49±3b	556±7d	206±3e	261±5e	158±4e	1431±139a	59±1d	194±1e	49±5e	565±45d	796±37ab	150±6a	94±3bc	4567±162d
	RN	45±2b	629±12c	251±9d	316±6d	194±6d	1611±165a	76±3cd	217±7d	65±6d	652±10bc	751±44b	111±17a	94±8bc	5011±232cd
No.4	CK	64±1a	1033±7a	638±2a	785±9a	888±11a	1043±38abc	195±14a	796±1a	225±5a	612±14b	713±14a	166±20a	150±3b	7308±90a
	BN70%	54±3ab	624±7b	347±11b	462±29b	468±8b	1131±104ab	74±5c	164±4e	145±6b	1102±9a	654±9b	150±5ab	166±6a	5541±116b

	BN95%	51±5b	490±6e	221±3e	357±4de	216±6d	1200±107a	101±10b	247±6d	67±8d	588±3bc	495±28c	121±16b	75±9c	4228±115c
	BN	59±6ab	600±11c	291±8c	396±5c	258±8c	984±37bc	83±7bc	302±8b	90±5c	616±20b	463±11cd	61±3c	73±7c	4278±118c
	YN	56±7ab	589±7cd	240±1d	339±2e	214±3d	1033±21abc	67±7c	270±4c	84±3c	590±8bc	479±20cd	44±9c	82±2c	4085±34c
	RN	61±6ab	572±5d	235±3de	386±4cd	239±6c	948±30c	67±5c	298±13b	86±8c	582±6c	443±18d	50±7c	70±3c	4036±18c
No.5	CK	57±4a	925±33a	729±39a	890±44a	1477±87a	712±92a	317±13a	874±38a	317±18a	540±28a	322±40a	101±4a	103±9a	7364±324a
	BN70%	37±2c	679±5b	375±4b	678±10b	671±6b	736±168a	133±3b	526±4b	171±7b	543±19a	307±27a	80±9ab	66±1b	5000±132b
	BN95%	47±2b	466±10d	297±3c	516±10c	567±41b	677±109a	120±6bc	357±11c	135±7c	530±15a	204±22b	68±16bc	43±2c	4027±127c
	BN	47±3b	609±8c	306±5c	508±10c	425±11c	774±54a	97±6cd	373±7c	125±4c	557±13a	250±34ab	54±3cd	51±1c	4177±85c
	YN	43±3bc	372±3e	178±2d	318±2d	209±4d	608±78a	63±13e	212±2d	62±2d	435±12b	199±9b	35±4d	32±2d	2764±74d
	RN	47±0b	360±4e	203±3d	317±4d	249±1d	551±59a	73±18de	180±7d	52±3d	398±5b	104±16c	62±8bc	8±1e	2605±60d
Wanghai 1															
No.1	CK	72±4a	358±15a	180±9a	814±45a	430±12a	1477±108a	143±10a	155±10a	73±4a	448±62a	152±14a	73±14a	33±2a	4408±182a
	BN70%	51±3cd	181±2b	81±4b	482±11b	230±7b	1263±50b	72±1b	58±2b	31±3b	281±4bc	70±3b	24±1b	18±0c	2842±74b
	BN95%	60±2b	112±4d	43±3c	222±4de	96±3d	1301±35b	30±2c	28±3d	21±4c	239±6bc	67±8b	22±0b	20±1bc	2260±33d
	BN	55±1bc	170±3bc	80±5b	318±5c	184±6c	1292±65b	51±8bc	42±5c	30±1b	313±29b	83±14b	39±3b	22±2b	2679±74bc
	YN	55±1bc	156±7c	48±4c	268±2cd	166±5c	1304±56b	49±3c	35±3cd	26±3bc	258±6bc	81±4b	42±13b	23±1b	2512±57c
	RN	46±2d	175±3bc	46±2c	187±1e	94±7d	1170±5b	41±14c	35±3cd	24±2bc	227±1c	87±18b	40±1b	21±2bc	2192±38d
No.2	CK	71±3a	337±15a	151±7a	881±10a	392±6a	1274±54a	102±8a	145±6a	65±8a	429±14a	132±6a	77±12a	31±3a	4087±105a
	BN70%	41±3c	205±10b	73±4b	440±6b	166±13b	1024±19c	36±3b	63±1b	28±2b	261±4d	81±13b	25±2b	19±1c	2462±50b
	BN95%	51±3b	129±4d	37±1c	197±3e	75±2e	1138±31bc	18±4c	24±1d	23±2b	254±1d	75±9b	31±9b	21±3bc	2072±39d
	BN	43±1c	143±6d	36±1c	172±13e	84±11de	1206±72ab	26±5bc	25±3d	23±1b	257±6d	75±5b	41±25b	20±3bc	2151±90cd
	YN	52±3b	186±10bc	65±3b	320±10c	130±6c	1108±32bc	35±6b	40±5c	29±3b	376±14b	93±7b	41±3b	21±4bc	2498±57b
	RN	43±2c	168±5c	45±2c	254±11d	104±8d	1132±27bc	30±8bc	36±3c	25±1b	304±2c	102±17b	49±7ab	26±2ab	2320±24bc
No.3	CK	65±1a	283±9a	169±4a	948±20a	427±11a	1291±42a	70±4a	97±5a	53±4a	622±16a	79±3a	49±1a	27±2a	4177±83a
	BN70%	43±3d	168±3b	73±2b	420±6b	184±2b	1299±93a	32±2c	34±5b	30±5b	483±18b	66±3b	30±3c	15±1c	2876±69b
	BN95%	51±3bc	57±2e	20±2d	110±2e	73±2e	1101±20b	20±1d	10±1d	14±2d	325±22d	32±1d	13±0e	21±1b	1848±40e
	BN	53±3b	129±1c	56±4c	255±12c	125±6c	1303±70a	39±2b	22±2c	23±2bc	403±7c	62±5b	24±1d	20±1b	2513±86c
	YN	44±2d	79±7d	10±2e	124±7e	80±1e	1070±16b	17±1d	13±1d	21±2cd	333±14d	47±6c	25±2cd	19±1b	1883±17e

	RN	47±1cd	88±3d	19±1d	176±1d	97±2d	1206±87ab	18±2d	15±2cd	20±1cd	365±14cd	69±4ab	41±4b	20±1b	2180±92d
No.4	CK	64±6a	301±8a	222±3a	1021±15a	517±12a	972±20b	89±8a	127±3a	82±1a	451±15b	70±7a	44±3a	27±1a	3987±57a
	BN70%	38±1c	161±4b	120±7b	515±22b	234±11b	1065±5a	34±2b	39±5b	35±4b	501±20a	34±5b	13±2b	10±1c	2800±71b
	BN95%	48±1b	55±2e	43±1d	194±1c	101±3cd	889±16c	18±5c	14±1cd	14±1c	354±3cd	23±2c	10±0b	16±2b	1779±27d
	BN	43±1bc	94±0c	62±3c	186±5c	113±5c	992±18b	23±4bc	20±2c	19±4c	389±13c	23±3c	13±1b	11±1c	1990±41c
	YN	43±2bc	65±5de	29±3e	121±3d	80±2e	839±23d	15±5c	15±2cd	16±1c	308±17e	28±1bc	12±3b	14±1bc	1586±17e
	RN	43±2bc	75±2d	38±5de	127±2d	87±1de	877±13cd	15±2c	13±0d	16±2c	339±16de	30±0bc	13±2b	11±1c	1684±9de
No.5	CK	80±8a	400±30a	453±47a	1655±146a	1136±106a	794±55a	152±22a	154±20a	137±21a	510±54a	39±8a	59±6a	23±1a	5593±515a
	BN70%	33±2b	121±1b	110±3b	786±14b	396±10b	581±27bc	47±6b	30±1bc	28±5b	265±7b	10±1b	13±1b	5±0c	2426±23b
	BN95%	36±1b	18±2d	11±1c	73±3d	50±4d	589±13bc	12±2c	8±1c	9±2b	179±3d	11±3b	4±1b	8±1b	1008±17e
	BN	42±2b	96±5b	90±1b	404±21c	216±10c	651±45b	26±2bc	28±1bc	27±3b	290±5b	12±1b	13±2b	7±3bc	1902±8bc
	YN	36±4b	34±4cd	21±2c	107±3d	74±3d	547±6c	14±4c	12±2c	13±2b	196±6cd	10±1b	6±3b	6±1bc	1076±15de
	RN	38±3b	53±6c	29±5c	237±3d	122±7cd	671±24b	20±8bc	86±62ab	48±27b	257±12bc	10±1b	11±9b	8±1bc	1590±46cd
Jingning 1															
No.1	CK	126±3a	683±7a	495±2a	73±1a	722±11a	133±11a	179±17a	321±3a	163±29a	692±38a	647±35a	99±22a	166±4a	4499±65a
	BN70%	98±3b	581±8b	414±8b	55±3b	484±7c	92±11ab	114±12b	240±9b	123±5b	561±12b	564±86ab	54±10b	149±8a	3529±42b
	BN95%	98±3b	468±9d	360±12c	45±1c	517±14b	121±24ab	117±13b	164±6d	88±5c	552±16b	519±55ab	66±6ab	119±8b	3234±37c
	BN	78±6c	490±14c	337±11d	35±1d	269±15e	88±17b	81±9c	182±8c	87±2c	512±25b	490±48b	42±19b	161±9a	2853±46d
	YN	83±3c	439±2e	318±7de	35±3d	329±11d	92±20ab	100±3bc	169±6cd	82±6c	538±27b	463±27b	45±6b	151±7a	2845±101d
	RN	86±2c	490±3cd	313±4e	38±2d	241±3e	81±6b	88±7bc	174±5cd	77±3c	556±17b	506±31b	42±11b	166±9a	2857±65d
No.2	CK	129±3a	717±16a	567±14a	73±2a	734±30a	103±10a	120±12a	398±13a	218±9a	790±19a	651±77a	90±21a	194±3a	4785±125a
	BN70%	103±2b	592±9b	439±10b	63±0b	479±12b	124±14a	110±22ab	288±6b	158±6b	719±20bc	604±51a	83±18ab	199±5a	3961±140b
	BN95%	90±2c	381±7d	324±6d	43±1d	310±2cd	106±5a	75±9bc	178±3cd	99±4c	650±3d	562±112a	49±6b	193±6a	3061±127e
	BN	93±1c	436±40c	354±4c	44±2d	322±3c	130±11a	70±18bc	184±3cd	105±0c	709±9c	577±51a	70±16ab	207±3a	3300±77de
	YN	104±2b	417±1cd	320±6d	43±2d	279±7d	130±27a	61±18c	171±7d	108±13c	758±11ab	616±87a	52±4ab	201±5a	3260±25de

	RN	111±6b	419±10cd	341±15cd	51±2c	342±9c	140±12a	69±6bc	192±7c	105±5c	781±27a	663±96a	76±9ab	200±17a	3490±38c
No.3	CK	136±3a	622±5a	497±4a	88±4a	741±17a	139±5c	69±5a	322±5a	158±3a	911±15a	691±82ac	136±5a	189±5a	4700±118a
	BN70%	112±2c	372±9b	321±6b	67±1b	427±12b	141±10c	53±5ab	181±2b	110±14b	884±8a	697±39abc	52±26ab	182±5a	3601±21b
	BN95%	96±3d	268±5e	228±4d	43±1d	270±1e	145±9c	36±5b	105±3d	78±3cd	757±15c	549±40c	38±4b	177±6a	2790±38d
	BN	107±3c	305±3d	282±3c	48±1c	347±6c	158±7c	67±8ab	114±5c	81±10cd	822±11b	599±82bc	95±12ab	154±3b	3178±81c
	YN	109±2c	313±6d	220±4d	46±1cd	222±3f	223±14a	40±3ab	93±0e	61±5d	785±28bc	716±45ab	45±5ab	158±5b	3031±88cd
	RN	120±3b	343±15c	278±11c	49±1c	307±5d	196±5b	60±26ab	123±3c	83±2c	919±29a	795±26a	106±81ab	190±5a	3568±194b
No.4	CK	130±2a	637±5a	651±7a	83±2b	1096±14a	132±22bcd	143±21a	372±9b	280±6a	847±17a	565±22b	96±24ab	137±2a	5169±98a
	BN70%	109±1ab	387±33b	384±2b	66±1c	580±3b	174±37bc	84±8b	224±3c	154±1b	781±2b	607±48b	65±2bc	144±1a	3760±50b
	BN95%	91±2b	204±1e	205±3e	37±0e	331±2d	112±25cd	45±3c	98±2d	75±1d	586±3d	323±54cd	40±3cd	85±5c	2232±24c
	BN	48±2c	617±5a	320±4c	53±0d	456±3c	96±5d	79±6b	449±3a	106±2c	379±7f	860±44a	121±39a	60±6d	3645±95b
	YN	100±1ab	256±5d	216±5d	35±2e	250±4e	185±15b	42±13c	97±4d	68±1d	632±10c	388±20c	24±1cd	105±4b	2398±24c
	RN	48±29c	341±9c	154±0f	115±2a	81±3f	623±26a	32±12c	104±20d	70±21d	490±27e	255±37d	10±1d	42±9e	2366±123c
No.5	CK	138±5a	631±5a	714±10a	123±1a	1730±33a	159±21a	257±11a	377±5a	286±5a	774±17a	366±30bc	129±23a	80±2a	5763±38a
	BN70%	98±3b	373±42b	338±1b	70±4b	643±11b	136±15a	111±25b	205±1b	128±1b	628±7b	479±9a	66±2b	96±4ab	3371±64b
	BN95%	85±5c	201±9d	221±6d	44±1d	514±11c	86±17bc	77±8bc	112±2d	86±3c	505±12d	273±69c	39±6bcd	56±3b	2298±111d
	BN	70±3d	138±8e	182±2e	26±2e	303±11e	81±10c	54±10c	59±6e	54±1d	329±3e	115±10d	29±4cd	99±40ab	1540±65e
	YN	71±4d	147±2e	150±4f	38±6d	251±2f	78±7c	58±16c	49±8e	43±9d	320±2e	153±13d	21±8d	111±16ab	1491±20e
	RN	92±3bc	262±10c	257±4c	54±3c	451±7d	121±11ab	70±15bc	132±4c	89±1c	538±6c	382±34b	58±6bc	66±4ab	2571±42c
Zhonghuang 2															
No.1	CK	94±7a	953±49a	500±34a	918±66a	790±55a	899±112a	258±11a	479±31a	100±6a	253±17b	219±16ab	73±5a	28±2a	5562±303a
	BN70%	83±7abc	675±46b	321±20b	682±43b	486±30b	930±93a	171±28b	301±18b	57±3b	251±17bc	239±26a	73±15a	25±1bc	4292±323b
	BN95%	74±2c	410±9c	204±7d	507±6c	344±6cd	824±33a	106±5d	184±6d	45±4bcd	243±6bc	182±6b	41±6a	20±2c	3184±72c
	BN	74±3c	441±13c	214±8d	470±17c	304±11d	805±92a	114±17d	181±4d	41±4cd	215±12c	205±14ab	50±12a	21±1cd	3135±181c
	YN	80±1bc	448±5c	203±3d	486±3c	306±5d	886±26a	103±35d	174±1d	38±6d	223±14bc	174±10b	42±17a	20±1c	3186±37c

	RN	88±1ab	593±47b	265±3c	614±20b	411±13c	966±44a	116±6cd	238±5c	52±6bc	296±9a	239±26a	67±12a	23±1cd	3970±122b
No.2	CK	91±3a	1064±8a	566±7a	1036±11a	875±11a	826±44a	235±8a	563±4a	124±3a	271±6a	227±19a	68±10a	28±6a	5973±77a
	BN70%	86±4a	907±1b	437±5b	867±10b	596±8b	908±22a	141±18b	387±6b	81±11b	279±6a	205±20a	49±8ab	26±1a	4969±22b
	BN95%	66±1bc	502±7e	222±5e	537±6de	311±5c	892±30a	75±10c	214±5d	45±5c	243±10bc	191±19a	50±11ab	21±2a	3370±53d
	BN	64±2c	601±11d	250±10d	508±8e	287±9d	845±47a	80±8c	194±7e	38±5c	222±10c	194±32a	42±9b	23±1a	3346±113d
	YN	74±5b	636±27d	260±11cd	560±20cd	325±13c	900±37a	103±12bc	219±9d	42±6c	238±10bc	182±17a	43±4b	21±1a	3602±126c
	RN	70±3bc	689±12c	276±6c	575±11c	332±4c	833±29a	100±23c	243±4c	50±3c	261±10ab	201±12a	63±3ab	24±1a	3716±47c
No.3	CK	141±6a	994±5a	531±4a	920±8a	707±8a	748±35b	176±2a	425±10a	100±15a	262±21b	184±10a	46±1ab	28±1a	5262±43a
	BN70%	142±4a	738±14b	324±5b	744±15b	449±4b	950±41a	97±3b	285±1b	69±2b	285±6ab	187±11a	49±3ab	25±1ab	4342±54b
	BN95%	126±5b	461±2e	163±3e	423±2e	222±4d	1008±104a	71±6cd	127±5d	38±1c	281±3ab	167±7ab	35±5c	21±2b	3144±115e
	BN	142±5a	554±6d	205±5d	446±4d	234±1d	956±37a	67±5de	137±2d	33±9c	277±14ab	174±12a	56±6a	21±1b	3301±49de
	YN	143±3a	551±10d	221±9c	506±10c	273±9c	997±51a	81±7c	165±8c	43±3c	281±8ab	143±4bc	34±4c	22±2b	3461±96c
	RN	137±7ab	593±5c	214±2cd	491±2c	260±2c	895±46ab	56±3e	166±7c	42±1c	301±3a	135±12c	43±1bc	24±1ab	3358±51c
No.4	CK	45±6a	1133±13a	599±10a	1014±16a	858±19a	793±44a	263±15a	541±14a	130±2a	277±6a	188±14a	74±10a	29±1a	5944±120a
	BN70%	37±1a	668±18b	307±12b	758±25b	478±18b	741±70a	108±13b	254±7b	51±5b	206±3b	72±23b	36±4b	15±1b	3732±140b
	BN95%	39±3a	372±35d	153±2d	443±4e	241±3e	732±21a	44±3c	135±5d	18±1d	172±11c	64±9b	15±7c	14±1b	2442±61d
	BN	44±2a	503±44c	224±4c	480±11de	255±2de	816±53a	67±7bc	149±10cd	25±3d	187±3bc	82±17b	36±2b	16±1b	2884±81c
	YN	46±5a	502±36c	222±7c	503±14cd	300±5c	855±45a	81±20bc	170±7c	35±2c	182±8c	90±12b	21±4bc	16±1b	3022±137c
	RN	42±5a	553±12c	230±5c	523±7c	282±11cd	855±27a	75±25bc	165±6c	35±1c	205±9b	86±10b	20±1c	16±1b	3088±10c
No.5	CK	89±3a	930±8a	506±5a	967±13a	863±10a	756±89a	302±14a	450±7a	116±2a	240±4a	116±14a	69±30a	22±1a	5426±112a
	BN70%	87±3ab	504±12b	294±13b	735±18b	604±13b	607±53abc	113±6b	183±6b	51±10b	170±7c	40±8c	19±3b	11±1bc	3419±37b
	BN95%	84±2ab	234±9e	132±3e	404±8d	285±5e	527±83c	60±12d	86±2d	10±3d	146±4d	41±3c	24±2b	9±1c	2042±77d
	BN	87±2ab	417±7c	225±1c	580±6c	380±5d	725±6ab	90±1bcd	147±5c	39±2bc	190±3b	84±30ab	34±13ab	12±0b	3008±58c
	YN	79±6bc	267±11d	167±0d	403±9d	303±4e	498±40c	69±26cd	88±6d	19±12cd	126±7e	34±4c	16±4b	9±0c	2077±114d

RN	75±1c	396±10c	236±3c	581±7c	423±10c	587±30bc	105±4bc	170±4b	43±8b	160±7cd	50±10bc	29±20ab	10±0bc	2865±79c

a Abbreviation: M-gal-rha-glu: Myricetin glucosyl-rhamnosyl-galactoside; M-gal: Myricetin galactoside; M-glu: Myricetin glucoside; Q-gal-rha-glu: Quercetin glucosyl-rhamnosyl-galactoside; Q-glu-rha-glu: Quercetin glucosyl-rhamnosyl-glucoside; Q-glu-rha-rha: Quercetin rhamnosyl-rhamnosyl-glucoside; Q-glu-rha: Quercetin rhamnosyl-glucoside; Q-gal: quercetin galactoside; Q-glu: Quercetin glucoside; K-glu-rha-glu: Kaempferol glucosyl-rhamnosyl-glucoside; K-gal: kaempferol galactoside; K-glu-rha: Kaempferol rhamnosyl-glucoside; K-glu: Kaempferol glucoside; TFG: Total flavonol glycosides; BN70%: samples under black net 70% shade treatment; BN95%: samples under black net 95% shade treatment; BN: samples under blue net shade treatment; YN: samples under yellow net shade treatment; RN: samples under red net shade treatment. For the same tea cultivars with the same serial number, data with different alphabetic letters (a, b, c, d, e, f) in a same row were significantly different at $P<0.05$. Significant difference analysis was carried out by the SAS System for Windows version 8.1 (SAS Institute Inc., Cary, NC, USA) using Tukey test. Data expressed as mean± SD represent n = 3 replicates.

[b] Relatively quantified by the corresponding aglycone.
